# Transcriptomic Analysis of *Trichoderma atroviride* Overgrowing Plant-Wilting *Verticillium dahliae* Reveals the Role of a New M14 Metallocarboxypeptidase CPA1 in Biocontrol

**DOI:** 10.3389/fmicb.2019.01120

**Published:** 2019-05-27

**Authors:** María E. Morán-Diez, Irene Carrero-Carrón, M. Belén Rubio, Rafael M. Jiménez-Díaz, Enrique Monte, Rosa Hermosa

**Affiliations:** ^1^Department of Microbiology and Genetics, Spanish-Portuguese Institute for Agricultural Research (CIALE), University of Salamanca, Salamanca, Spain; ^2^College of Agriculture and Forestry (ETSIAM), University of Córdoba, Córdoba, Spain; ^3^Institute for Sustainable Agriculture (IAS), Spanish National Research Council (CSIC), Córdoba, Spain

**Keywords:** Verticillium wilt, mycoparasitism, microarray, carboxypeptidase, secondary metabolism, *cpa1*-overexpressed mutants

## Abstract

*Verticillium dahliae*, a vascular-colonizing fungus, causes economically important wilt diseases in many crops, including olive trees. *Trichoderma* spp. have demonstrated an effective contribution as biocontrol agents against this pathogen through a variety of mechanisms that may involve direct mycoparasitism and antibiosis. However, molecular aspects underlaying *Trichoderma*–*V. dahliae* interactions are not well known yet due to the few studies in which this pathogen has been used as a target for *Trichoderma*. In the present study, *Trichoderma atroviride* T11 overgrew colonies of *V. dahliae* on agar plates and inhibited growth of highly virulent defoliating (D) *V. dahliae* V-138I through diffusible molecules and volatile organic compounds produced before contact. A *Trichoderma* microarray approach of T11 growing alone (CON), and before contact (NV) or overgrowing (OV) colonies of V-138I, helped to identify 143 genes that differed significantly in their expression level by more than twofold between OV and CON or NV. Functional annotation of these genes indicated a marked up-regulation of hydrolytic, catalytic and transporter activities, and secondary metabolic processes when T11 overgrew V-138I. This transcriptomic analysis identified peptidases as enzymatic activity overrepresented in the OV condition, and the *cpa1* gene encoding a putative carboxypeptidase (ID number 301733) was selected to validate this study. The role of *cpa1* in strain T11 on antagonism of V-138I was analyzed by a *cpa1*-overexpression approach. The increased levels of *cpa1* expression and protease activity in the *cpa1*-overexpressed transformants compared to those in wild-type or transformation control strains were followed by significantly higher antifungal activity against V-138I in *in vitro* assays. The use of *Trichoderma* spp. for the integrated management of plant diseases caused by *V. dahliae* requires a better understanding of the molecular mechanisms underlying this interaction that might provide an increase on its efficiency.

## Introduction

The use of *Trichoderma* species for the biocontrol of plant diseases has been related mainly to their antagonistic abilities against phytopathogenic fungi and oomycetes (Howell, 2003). Thus, the mechanisms of *Trichoderma-*based plant disease biocontrol rely mainly on the production of antibiotics and/or hydrolytic enzymes as well as competition for nutrients (Lorito et al., 2010).

Functional characterization of individual *Trichoderma* genes has provided valuable insight into mycoparasitism of fungal pathogens by *Trichoderma* spp. and their role in the biocontrol of plant diseases. Although a comparative genomics study has shown that mycoparasitism is an ancestral property of the genus *Trichoderma* (Kubicek et al., 2011), there are still major gaps in our understanding of the molecular determinants responsible for triggering and regulating that mechanism. A recent study has suggested that lateral gene transfer is linked to the ability of *Trichoderma* to parasitize taxonomically related fungi and this may have allowed *Trichoderma* fungi to modify their lifestyles (Druzhinina et al., 2018). High-throughput approaches have been undertaken in studies on mycoparasitism by *Trichoderma* spp. (Samolski et al., 2009; Seidl et al., 2009; Reithner et al., 2011; Atanasova et al., 2013; Steindorff et al., 2014). A comparative transcriptomics study using *Rhizoctonia solani* as a target fungal pathogen has confirmed the bigger mycoparasitic potential of *Trichoderma atroviride* and *Trichoderma virens* as compared to that of *Trichoderma reesei*, and also noted different mycoparasitic strategies existing in *Trichoderma* spp. (Druzhinina et al., 2011). Following an RNA-seq approach, mycoparasitism-related genes of *Trichoderma harzianum* have been identified in response to *Sclerotinia sclerotiorum* (Steindorff et al., 2014).

Recent studies have shown that *Trichoderma* spp. bear potential as biocontrol agents for the integrated management of Verticillium wilt (VW) of olive caused by *Verticillium dahliae*, which is a key strategy for the efficient management of this disease (Jiménez-Díaz et al., 2009, 2012; López-Escudero and Mercado-Blanco, 2011; European Food Safety Authority Panel on Plant Health, 2014; Carrero-Carrón et al., 2016, 2018). *V. dahliae* is a strict asexually reproducing Ascomycete that can survive in the soil by means of melanized microsclerotia without a host for at least 14 years and in addition to olive trees cause vascular diseases in more than 400 dicotyledonous plant species, including many agricultural and horticultural important crops and ornamental plants (Klosterman et al., 2009; European Food Safety Authority Panel on Plant Health, 2014). This fungal pathogen has a distinctly clonal population structure which comprises nine clonal lineages (Milgroom et al., 2014). Superimposed onto *V. dahliae* clonality are two types of pathogenic variation, namely defoliating (D) and non-defoliating (ND) pathotypes (symptom types) (Milgroom et al., 2014; Jiménez-Díaz et al., 2017). Members of the D pathotype cause defoliation of cotton, olive and okra only and are exclusively in lineage 1A, whereas those of the ND pathotype do not defoliate these plant species and are in any of all other lineages (Jiménez-Díaz et al., 2011; Milgroom et al., 2014). VW cannot be managed with a singly applied control measure because of the wide host range and long-term survival of the pathogen in soil, as well as the susceptibility of most olive cultivars to the highly virulent D pathotype. However, in spite of the importance of VW diseases and the potential of *Trichoderma* spp. as biocontrol agents, the interactions between *Trichoderma* spp. and *V. dahliae* have not been yet addressed in biocontrol studies at molecular level.

In the present study, diverse *in vitro* antagonism assays using as targets five strains of *V. dahliae* representative of different lineages, pathotypes and races (Jiménez-Díaz et al., 2017) showed that *T. atroviride* T11 has a high mycoparasitic and antibiotic potential against this pathogen. The transcriptomic changes in T11 in response to V-138I as well as T11 genes that might be involved in mycoparasitism were further examined using a *Trichoderma* microarray comprising 385,000 probes designed against the genomes of *T. atroviride*, *T. virens* and *T. reesei*. Real-time quantitative PCR (RTqPCR) supported the microarray-based evidence for differentially expressed *Trichoderma* genes in T11 during overgrowth of V-138I colonies. One of these genes, identified within the group of proteolysis-related genes, was used for further development of *cpa1*-overexpressed T11 transformants and to demonstrate the validity of the microarrays analysis. Results revealed a role of M14 metallocarboxypeptidase CPA1 in the antagonism of *T. atroviride* against V-138I in *in vitro* assays. Elucidation of the molecular mechanisms that govern the inhibition of *V. dahliae* by *T. atroviride* may help to better understand the interaction between the two fungi and to design novel strategies for the integrated management of VW in olive and other economically important crops.

## Materials and Methods

### Fungal Strains

*Trichoderma atroviride* IMI 352941 (CABI Bioscience, Egham, United Kingdom), referred to as T11, was used along this study. Previously typed *V. dahliae* strains V-T9, V-1477I, V-1900I, and V-138I (lineage 1A, D pathotype and race 2), and strain V-1558I (lineage 2A, ND pathotype and race 1) (Jiménez-Díaz et al., 2011, 2017) were used as a target in this experimental study. *V. dahliae* V-138I strain was also used in the microarray study and in *in vitro* assays aimed to test the biocontrol potential of wild-type and transformant strains of *T. atroviride*. Both, *T. atroviride* and *V. dahliae* strains were routinely cultured on potato dextrose agar medium (PDA; Difco-Becton, Dickinson, Sparks, MD, United States).

### Culture Conditions and *in vitro* Antagonism Experimental Procedures

Unless otherwise indicated, *V. dahliae* strains were grown at 25°C in the dark and 4 days in advance to *T. atroviride* T11 wild-type or transformants strains. Dual confrontations between *T. atroviride* T11 and the five *V. dahliae* strains were carried out as previously described (Rubio et al., 2009; Montero-Barrientos et al., 2011). Briefly, a 5-mm diameter agar plug colonized by strain T11 was placed at 2 cm from the border on the opposite side of the same plate on which an agar-plug colonized by *V. dahliae* was grown. Cultures of *V. dahliae* growing alone were used as controls. Dual cultures were performed in triplicate and pathogen colony diameters were measured after 12 days of incubation. Colony area (cm^2^) of each of the five *V. dahliae* strains was calculated and results were expressed as the percentage growth inhibition of *V. dahliae* by T11 with respect to the mean colony area of each fungus grown alone. A modified procedure to the one described above was used to measure the effect of volatile organic compounds (VOCs) on inhibition of the pathogen growth. Discontinuous agar cultures were designed by removing a 2.5-cm-wide strip of PDA medium from the Petri dishes before inoculation with the fungi. Cultures were incubated for up to 24 days and percentage growth inhibition was recorded.

Growth assays on cellophane sheets and 14 kDa-cut-off dialysis cellulose membranes were carried out in triplicate as previously described (Rubio et al., 2009). The diameters of the fungal colonies were measured after 10 days of incubation. Results were expressed as the percentage growth inhibition of each *V. dahliae* strain by *T. atroviride* T11 with respect to the mean colony area of each fungus grown alone. A similar culture method on cellophane-covered PDA dishes was used for confronting *T. atroviride* T11 against itself and against *V. dahliae* V-138I for microarrays assays. Mycelia of *T. atroviride* T11 were harvested when they had reached a position of ca. 5 mm from that of *V. dahliae* (near *V. dahliae*, NV) and after *T. atroviride* T11 had overgrown the *V. dahliae* colony (over *V. dahliae*, OV). Mycelia of *T. atroviride* T11 grown alone served as controls (CON). In addition to these conditions, mycelium from T11 confronted with itself (NT) was harvested for RTqPCR validations. Mycelia of strain T11 collected in these conditions were also used for RNA extraction as a previous step before the RTqPCR validation procedures and for protein extractions in the protease activity measured in T11 wild-type. Mycelia from four plates were pooled for RNA extraction of each individual condition followed by the cDNA synthesis and three biological replicates of each condition were considered for this study.

For *cpa1* gene expression, mycelia collected from T11 wild-type and from transformant strains were obtained following a two-step liquid culture approach (Cardoza et al., 2006). First, the strains were grown in potato dextrose broth (PDB, Difco-Becton) at 25°C and 200 rpm for 48 h. The fungal biomass was harvested, washed and transferred to minimal medium (MM) (Penttilä et al., 1987) with 0.5 or 2% glucose as the only carbon source, or 0.5% glucose MM medium supplemented with 0.5% V-138I cell walls that were obtained according to Fleet and Phaff (1974). After 24 h of incubation at 25°C and 200 rpm, mycelia were collected by filtration, thoroughly washed with sterile water, lyophilized, kept at -80°C and used for RNA extraction. Following a similar culture method, supernatants from T11 wild-type and *cpa1* transformants grown on MM supplemented with 2% glucose for 24 h were used for protein extraction followed by measurements of protease activity and antifungal activities. Mycelia and supernatants from three independent biological replicates were collected for RNA and protein extractions, respectively.

### DNA, RNA, and Protein Assays Procedures

Total fungal DNAs were extracted following the method of Raeder and Broda (1985), using mycelium collected from a PDB culture incubated at 25°C and 200 rpm for 48 h.

Total RNA from each independent biological replicate was extracted using TRIZOL^®^ reagent (Invitrogen Life Technologies, Carlsbad, CA, United States) following the manufacturer’s instructions, and the extracted RNA was treated with DNase I (Fermentas, Burlington, Canada). For microarray assays, DNase I-treated RNA was purified using the RNeasy MinElute Cleanup kit (Qiagen, Hilden, Germany) and cDNAs were synthesized, amplified and labeled by Roche-NimbleGen (Roche NimbleGen Inc., Madison, WI, United States). For RTqPCR assays, a total of 1 μg of RNA was reverse-transcribed into cDNA using the PrimeScript^TM^ RT reagent mix kit with an oligo (dT) primer in a final volume of 20 μl (Takara Inc., Tokyo, Japan). The synthesized cDNA was diluted with 80 μl of water and used as a template for RTqPCR reactions.

Protease activity was determined in a colorimetric assay by measuring the hydrolysis of azocasein at 366 nm, as previously described (Holwerda and Rogers, 1992; Montero-Barrientos et al., 2011). *Trichoderma* mycelia and supernatants were homogenized and mixed, respectively, in 100 mM Tris buffer, pH 7.5, at 4°C for 1 h and protein extracts were recovered by centrifugation at 12,000 × *g* at 4°C for 20 min. Quantitative protein determination was performed with the Bradford assay (Bradford, 1976), using bovine serum albumin as a protein standard. The reaction mixture (0.325 ml) containing 1% (w/vol) azocasein (Sigma-Aldrich Química S.A., Madrid, Spain) in 50 mM sodium acetate buffer, pH 5.5, and 10 μg of proteins or different volume (5 and 10 μl) from protein extracts, was incubated at 30°C for 1 h. Total activity corresponded to mmol of azocasein hydrolyzed in 1 min, and specific activity corresponded to mmol of azocasein hydrolyzed in 1 min per mg of protein. Assays were performed in triplicate and using, at least, three biological replicates. Data represent mean values with standard deviations.

### Isolation and Characterization of *cpa1*-Overexpressed Transformants

Plasmid pRF-HUE-CPA1 (10.28 kb) was constructed to express *cpa1* gene in *T. atroviride* T11 under the constitutive control of the glyceraldehyde 3-phosphate dehydrogenase promoter (PgpdA) of *Aspergillus nidulans.* A 1,576-bp fragment was amplified by PCR using primers 301733UserO3 (5′-GGACTTAAUATGAAGACTGTTCTTCCCTGGGC-3′) and 31733UserO4 (5′-GGGTTTAAUGCCGCATGAGAGACGCCCATT-3′), and genomic DNA from strain T11. This fragment included the entire 1,314-bp *cpa1* coding region and 262-bp of its native terminator. The pair of primers included the sequences needed to digest the PCR product with the USER^TM^ cloning technology in order to ligate it to pRF-HUE plasmid (8,709-bp) with 3′ overhangs generated by the combined cutting of *Pac*I and *Nt.Bbv*CI enzymes (Frandsen et al., 2008). pRF-HUE-CPA1 was used to genetically transform *T. atroviride* T11 employing *Agrobacterium tumefaciens* strain AGL-1 (Lazo et al., 1991) for *A. tumefaciens*-mediated transformation as previously described (Mullins et al., 2001; Cardoza et al., 2006). In parallel, strain T11 was also transformed with the vector-backbone pRF-HUE to obtain empty vector transformants to be used as a control. Transformants were selected for hygromycin resistance.

Expression of the *cpa1* gene and protease activity was evaluated as mentioned above. The antifungal activity of protein extracts of *cpa1*-overexpressed T11 transformants and T11 wild-type obtained as described above was tested against *V. dahliae* V-138I. A conidial suspension (200 conidia in 10 μl) of V-138I was added to wells of sterile 96-well flat-bottomed microtiter plates along with 10, 25, or 50 μl of filter-sterilized (0.22-μm syringe filter; Millipore) of *Trichoderma* protein extracts as described above. Antifungal activity of protein extracts previously boiled for 10 min were also tested. PDB medium was added to each well up to a final volume of 150 μl. Plates were incubated at 25°C and 20 rpm in the dark for 72 h. *V. dahliae* growth was determined at 0, 24, 48, and 72 h by measuring optical density at 595 nm using a Sunrise microtiter plate reader (Tecan Ibérica, Barcelona, Spain) after shaking for 5 s. Each assay was performed using six technical replicates and protein extracts from three independent fungal cultures.

### Microarray Design and Analysis

A *Trichoderma* microarray was constructed by Roche-NimbleGen using the unmasked FASTA file of the genomes of *T. atroviride*^1^, *T. virens*^2^, and *T. reesei*^3^. This microarray comprised 385,000 60-mer probes, which encompassed 11,643 genes of *T. atroviride*, 11,100 genes of *T. virens* and 9,129 genes of *T. reesei*.

The hybridizations and data acquisition were performed by Roche-NimbleGen, as previously reported for the *Trichoderma* high-density oligonucleotide (HDO) microarray v2 (Rubio et al., 2012). Digitization of the fluorescent signals emitted after the hybridization was performed using an Axon GenePix 4000B scanner with NimbleScan 2.3 software. Nine microarrays were examined (three replicates for each of NV, OV, and CON conditions) and the images obtained and raw probe intensity values were analyzed. A robust multichip average (RMA) convolution model was applied for background correction, and the corrected probe intensities were then normalized using a quantile-based normalization procedure (Irizarry et al., 2003). Finally, the processed data for the probes of each gene were summed to produce a measurement of expression. Following this, a multi-class significance analysis of microarray (SAM) test was carried out on the expression values using a fold-change (FC) > 2 and a false discovery rate (FDR) of 0.15 (*P* < 0.05) to identify genes displaying a significant difference in expression level. The analysis was performed using the FlexArray 1.6.1.1 program through the R software. Transcripts showing a significant differential expression were annotated according to gene ontology (GO) terms (Ashburner et al., 2000). A GO-term enrichment analysis was carried out with AgriGO, available at http://bioinfo.cau.edu.cn/agriGO/analysis.php. All unknown proteins were subjected to blastp searches to check whether any of them had been identified for another fungus besides the three *Trichoderma* spp. annotations, applying an *E*-value < 10^-20^ level. The proteins for which no function could be predicted were termed as unknown.

The microarray data are available at the GEO database with accession number GSE66835.

### Real-Time Quantitative PCR (RTqPCR)

A total of 16 genes were randomly selected among the differentially expressed genes in the transcriptomic study using microarrays for expression analysis by RTqPCR. Primers are given in Supplementary Table S1. Among those genes, 10 genes from a set of 143 genes differently expressed when T11 overgrew V-138I were analyzed. Samples representative of condition NT (T11 grown against T11) were used as control. The expression levels of *cpa1* gene were analyzed in mycelia collected from T11 wild-type and *cpa1* transformant strains grown under identical conditions as described above. The quality of all RNAs was determined in 1.5% agarose gels and the absorbance measurements (NanoDrop Spectrophotometer, Thermo Scientific, Wilmington, DE, United States) were used as an indicative of their purity with OD ratios of ∼ 2.0 for 280/260 and ∼2.0 or higher for 260/230. Reaction mixtures and amplification conditions were performed as previously described (Montero-Barrientos et al., 2011). Real-time PCR were performed using the cDNAs of four pooled plates and three biological replicates for each condition (OV, NV, NT, and CON) (microarray assays) and from three independent cultures for T11 wild-type and transformant strains (characterization of transformants). All PCRs were performed in triplicate on a StepOne Plus^TM^ device (Applied Biosystems, Foster City, CA, United States). Standard curves were measured for dilution series of pooled cDNA samples; the slope line of calibration and efficiency percentage of each primer pair were calculated using the Applied Biosystems software and they are given in Supplementary Table S1. Gene expression levels were calculated from the threshold cycle (CT) according to the 2^-ΔΔCT^ method (Livak and Schmittgen, 2001) using the α-actin transcript as an internal reference.

### Statistical Analyses

All data were collected from at least three independent replicates and expressed as the mean values. Statistical comparisons were evaluated by one-way ANOVA followed by a *post hoc* Tukey’s test using the Statview 5.0 software. Confidence intervals of 95% or 99% were set.

## Results

### Biological Control Potential of *Trichoderma atroviride* T11 Against *Verticillium dahliae*

The ability of T11 as an antagonist of *V. dahliae* strains differing in lineage, race and pathotype was tested under three experimental conditions: (i) direct confrontation in dual cultures, (ii) antibiosis triggered by non-volatile compounds (non-VOCs) such as hydrolytic enzymes or antibiotics, and (iii) antibiosis triggered by volatile organic compounds (VOCs).

In a dual confrontation assay, strain T11 was able to overgrow and sporulate on colonies of the five tested *V. dahliae* strains, reducing the colony area regardless their phylogeny, race and pathotype (Table 1). The largest colonies of *V. dahliae* strains developed in the control plates, and significant reductions occurring in colony area of the pathogen being indicative of the antagonistic ability of T11 against *V. dahliae* strains. The largest and least reduction occurred for strains V-T9 and V-1900I, respectively (Table 1).

**Table 1:** Inhibition (%) of colony area of five strains of *Verticillium dahliae* (V) in dual culture with *Trichoderma atroviride* T11 for 12 days, and by hydrolytic enzymes/metabolites secreted by strain T11 grown on cellophane or a 14-kDa cut-off dialysis membrane for 10 days.

	Dual culture^∗^	Cellophane	Dialysis membrane
V-1558I	66 ± 2.6 ab	100 ± 0.0 a	100 ± 0.0 a
V-1477I	57 ± 1.3 bc	95 ± 4.6 a	77 ± 5.1 b
V-1900I	48 ± 7.6 c	100 ± 0.0 a	100 ± 0.0 a
V-138I	57 ± 1.2 bc	90 ± 10.0 ab	100 ± 0.0 a
V-T9	68 ± 3.8 a	76 ± 4.5 b	94 ± 5.7 a


**^∗^V. dahliae strains were grown for 4 days before the sowing strain T11. Values are means of three replicates with the corresponding standard deviation. Values in the same column with different letters are significantly different according to Tukey’s test (P < 0.05)*.*

The antagonistic potential of non-VOCs of T11 against the five *V. dahliae* strains was evaluated in cellophane sheet and dialysis membranes by measuring the growth inhibition of the pathogen expressed as percentage of colony area in the control. Values are summarized in Table 1. The total extracellular compounds secreted by T11 displayed a marked growth inhibition of four out of the five *V. dahliae* strains, the largest reduction occurring for strains V-1558I and V-1900I. Compounds secreted by strain T11 of molecular weight less than 14-kDa also inhibited growth of the five pathogen’s strains, with the significantly (*P* < 0.05) lesser inhibition values occurring against strain V-1477I.

The antagonistic activity of T11 against D *V. dahliae* V-138I was also explored in a discontinuous agar system to determine whether VOCs might be involved in antagonism of this pathogen. T11 reduced the colony growth of V-138I at a rate of 23.0 ± 0.5% after 2 days of exposure under that growth condition, and growth inhibition was increased to 58.5 ± 15.0% after 7 days without the involvement of non-VOCs. Moreover, T11 growth was also temporarily inhibited by V-138I, although T11 finally jumped the gap in the medium and overgrew the *V. dahliae* mycelium (Supplementary Figure S1).

### Transcriptomic Changes in *Trichoderma atroviride* T11 Induced by *Verticillium dahliae* V-138I

**FIGURE 1:**
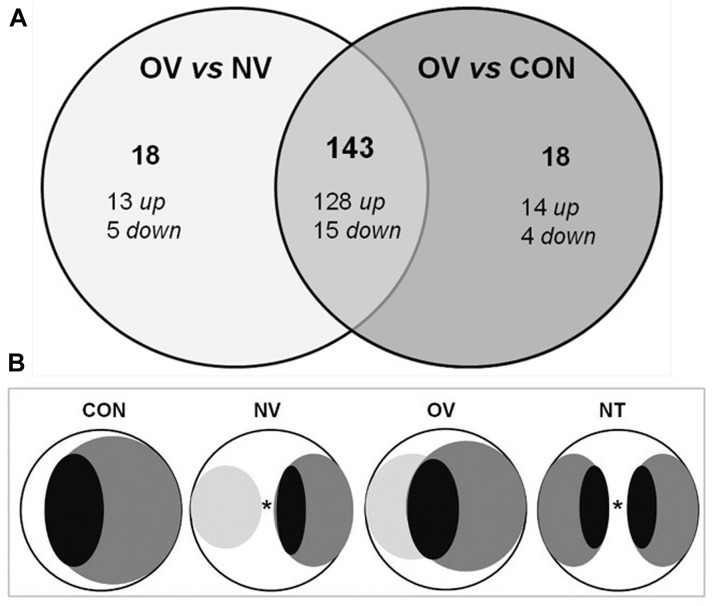
Microarrays data analysis and experimental setup of *Trichoderma atroviride* T11 against *Verticillium dahliae* V-138I. **(A)** Venn diagram of differentially expressed genes within the set of microarray data. Gene significance was assigned to more than twofold change (FDR: 0.15). Comparative analysis is showed for T11 overgrowing V-138I (OV) in comparison with those in T11 grown at 5 mm from V-138I (NV) and in T11 grown alone (CON). Total number of genes and their subgroups of up- and down-regulated genes are showed for each comparative analysis. The intersection between circles displays the total number of genes up- and down-regulated differently expressed under the overgrowing condition (OV). **(B)** Scheme of the experimental growth conditions designed for the microarrays. *T. atroviride* T11 and *V. dahliae* V-138I, that was inoculated 4 days in advance, were grown onto cellophane-covered PDA plates until samples were collected. T11 is drawn in dark gray color and V-138I is delineated in light gray color. Black oval dots represent the sampling areas that were collected for RNA extraction. CON, T11 grown alone; NV, T11 grown at 5 mm from V-138I; OV, T11 overgrowing V-138I; NT, T11 confronted with itself for RTqPCR validation. ^∗^5-mm space left between both organisms.

Two sets of genome-wide expression analyses were performed using a *Trichoderma* microarray of 385,000 probes to identify genes involved in mycoparasitism of *T. atroviride* T11 on D *V. dahliae* V-138I: (i) T11 overgrowing V-138I (OV) was compared to T11 growing alone (CON), and (ii) OV condition was compared to T11 that had grown up to ca. 5 mm from V-138I (NV) (see Figure 1B for more details on the experimental design and samples collection). The expression of a total number of 161 genes (0.47%) significantly (FDR, 0.15) expressed by more than twofold in condition OV compared to NV, and similarly occurred between condition OV and CON. Out of these 161 genes, 143 genes were identified as being differentially expressed in the OV condition (Figure 1A, Table 2 and Supplementary Table S2). Most of the differential expressions (98.6%) were identified from probes generated against the *T. atroviride* genome. Thus, at least these 143 genes can be associated with mycoparasitism potential. Among them, 128 genes were up-regulated (89.5%) and 15 genes were down-regulated (10.5%). By preliminary analysis, GO terms were assigned to 104 out of the 143 genes. Further enrichment analysis revealed that 30 GO categories were significantly overrepresented (*P* < 0.05) in T11 when it was overgrowing V-138I. Thereafter, the gene distribution in GO categories within the three main ontology categories – Biological Process (BP), Molecular Function (MF) and Cellular Component (CC) - was analyzed. Because of overlapping among several GO terms, we further examined whether the differentially expressed genes were associated with similar GO categories. “Metabolism” was overrepresented within the BP category (57 genes, *P*: 6.4 e^-5^) together with, and particularly, “proteolysis” (11 genes, *P*: 3.2 e^-5^). In the MF category, “catalytic” (71 genes, *P*: 2.1 e^-12^) and “transporter” (12 genes, *P*: 0.0014) activities were also overrepresented. The genes with catalytic activity that were most differentially expressed corresponded to oxidoreductases, hydrolases, and ligases. The CC category “integral to membrane” was also overrepresented, with 11 genes (*P*: 3.5 e^-8^). In addition, proteins from the 39 genes for which the GO term was not assigned were subjected to a blastp search. Putative function was predicted for 11 of those 39 genes, whereas no function was found for the remaining 28 proteins (Table 2). To overcome problems derived from the automatic annotation (overlaps and no GO-term assignation), a blastp search was performed with proteins of the 143 differentially expressed genes. As a result, the functional distribution of such genes within physiological events was recorded separated into three groups: metabolism, cellular processes and signaling, and information storage and processing (Supplementary Table S2). Data revealed a marked up-regulation of the transcripts involved in the primary metabolic processes of carbohydrates (21 CAZyme genes), proteins (10 genes), lipids and fatty acids (seven genes), and amino acids (four genes). The main hydrolytic activities related to mycoparasitism corresponded to glucanases and peptidases. Up-regulation of transcripts involved in secondary metabolic processes was also observed, with oxidoreductases and monooxygenases being the most abundant, with seven genes each. Within cellular processes and signaling events, transport (14 genes), defense (6 genes), and signaling (4 genes) were up-regulated and were the most represented.

**Table 2:** Summary of the functional distribution of the 143 differentially expressed genes during the overgrowth of *Trichoderma atroviride* T11 on *Verticillium dahliae* V-138I.

	No.	
	
Probe set	Up-regulated	Down-regulated
**Metabolism**		
Carbohydrate	21	2
Lipid and fatty acid	7	1
Protein	10	–
Amino acid	4	–
Nucleic acid	2	–
Secondary	20	2
Energy	5	1
**Cellular processes and signaling**		
Transport	14	–
Defense	6	–
Signaling	4	2
Cell–cell contact and communication	1	1
Detoxification	1	–
Cell wall and membrane	2	–
Regulation	3	–
Post-translation events	3	–
**Information storage and Processing**		
Transcription	1	–
Translation	2	–
Unknown function	22	6


**FIGURE 2:**
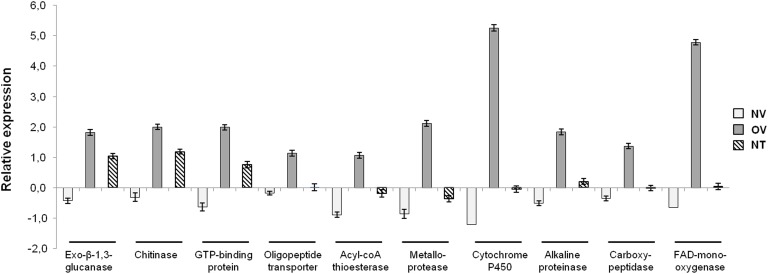
RTqPCR expression analysis of ten JGI-referred genes of set of 143 genes differently expressed when *T. atroviride* T11 overgrows *V. dahliae* V-138I (OV). Expression levels were obtained by comparing the expression of these genes in T11 grown at 5 mm from V-138I (NV), T11 grown at 5 mm from itself (NT) and T11 grown alone (CON) conditions. Identification in the *T. atroviride* genome (JGI) is as follows: exo-β-1,3-glucanase (48371), chitinase (52592), GTP-binding protein (224184), oligopeptide transporter (232557), acyl-CoA thioesterase 2 (282317), metalloprotease (179435), cytochrome P450 monooxygenase (295844), alkaline proteinase (302419), carboxypeptidase A1 (301733), and FAD-monooxygenase (32449). Ct values were referred to the CON condition as a basal reference. Data are the mean of three biological replicates and are displayed as the log10 of the relative quantity (RQ, 2^-ΔΔCt^) of target genes compared with the quantity of actin gene used as a reference.

To validate the microarray results, RTqPCR assays were performed using 10 arbitrarily chosen genes within the set of the 143 genes differentially expressed in the OV condition (Figure 2). In addition to the three conditions evaluated in the microarray (CON, NV, and OV), we included a fourth condition whereby T11 was grown up to a distance of 5 mm from itself (NT) (Figure 1B). RTqPCR profiles confirmed the results obtained in the microarray analysis for all genes evaluated, the highest expression level of the 10 genes analyzed occurring for those detected in the OV condition (Figure 2). An additional RTqPCR assay was done with two genes selected from the set of 18 genes differently expressed when comparing conditions OV and CON (Supplementary Figure S2A) and with four out of the 18 genes obtained from comparison between conditions OV and NV (Supplementary Figure S2B). Similar results were obtained when including the NT condition in the analysis (data not shown). The hit description and distribution in physiological processes of the two 18-gene sets from both comparisons (OV vs. CON and OV vs. NV) are given in Supplementary Tables S3, S4, respectively. Up-regulated hydrolase (two genes) and transporter (three genes) activities and down-regulated secondary metabolism activity (two genes) were observed among 15 genes to which function could be assigned in the OV vs. CON conditions. Likewise, the 18 genes (13 up- and 5 down-regulated) showing differential expression between OV and NV conditions formed a separate group (see Supplementary Table S4 in Supplementary Material) in which transporter activity was the most widely represented (four genes), and two out of these four genes were down-regulated. RTqPCR analyses were performed using, respectively, two and four genes chosen arbitrarily from the two sets of 18 genes of each comparison (Supplementary Figure S2). These six genes (five up- and one down-regulated) showed expression profiles compatible with a mycoparasitic activity since their highest or lowest expression levels corresponded to the OV condition.

Since proteolysis was one of the overrepresented catalytic activities within the set of genes differently expressed when T11 overgrew V-138I (condition OV), we analyzed this enzymatic activity in the OV, NV, and CON conditions. Value obtained in OV (36.75 ± 0.54 mmol/min per mg protein) was significantly higher than that recorded in NV (32.05 ± 1.54) or CON (28.92 ± 1.17), with no significant differences between activities in the last two conditions.

### Analysis of *cpa1* Gene and Characterization of *cpa1*-Overexpressed Transformants *in vitro*

One of the genes in the proteolysis group that was identified as being differentially expressed in condition OV compared with conditions NV, NT, and CON, both in microarray and RTqPCR analyses, was selected for further studies in this present work. A search for the protein ID number, assigned to as 301733, at the JGI Genome Portal Search application within the *T. atroviride* IMI 206040 database, returned a result that matched with a putative carboxypeptidase (CPA, from now on noted as CPA1) that belongs to the M14 family of metallocarboxypeptidases. The available *T. atroviride* IMI 206040 genome was the starting point to obtain the *cpa1* sequence. Two oligonucleotides, which contained a sequence target for *Pac*I and *Nt.Bbv*CI enzymes, respectively, were designed over *cpa1* sequence to amplify a 1,567-bp fragment from *T. atroviride* T11 genomic DNA by PCR analysis. This PCR fragment, which contains the *cpa1* coding region plus 262-bp of its terminator, was sequenced and showed 100% sequence identity with that of *T. atroviride* IMI 206040 but did not contain any intron as determined when cDNA was used as a template. The expression analysis of *cpa1* gene in T11 wild-type grown under different media conditions showed that the highest expression occurred in MM supplemented with 0.5% V-138I cell walls. This expression had a 5.3- to 5.8-fold difference relative to expression in MM supplemented with 0.5% glucose used as a reference control. No significant differences, relative to control, were obtained in MM supplemented with 2% glucose (0.85 to 1.26-fold change).

In order to characterize the *cpa1* gene functionally and its potential role on biocontrol of *V. dahliae* V-138I by *T. atroviride* T11, plasmid pRF-HUE-CPA1 was constructed and transformed in strain T11 (Supplementary Figure S3). Transformants were selected through five rounds of hygromycin resistance on PDA plates, with the fourth round being under non-selective media in order to obtain genetic stability of fungal cells. From a total number of 62 colonies obtained in the first round of selection, only two, namely *cpa1*-6.3 and *cpa1*-6.7, showed resistance on selective media in the final round followed by several rounds of selection of monosporic cultures. A 1,500-bp PCR product confirmed the presence of the pRF-HUE-CPA1 vector in the genome of the two transformants. One transformant (TaTC-0) containing the vector-backbone pRF-HUE was used as transformation control.

**FIGURE 3:**
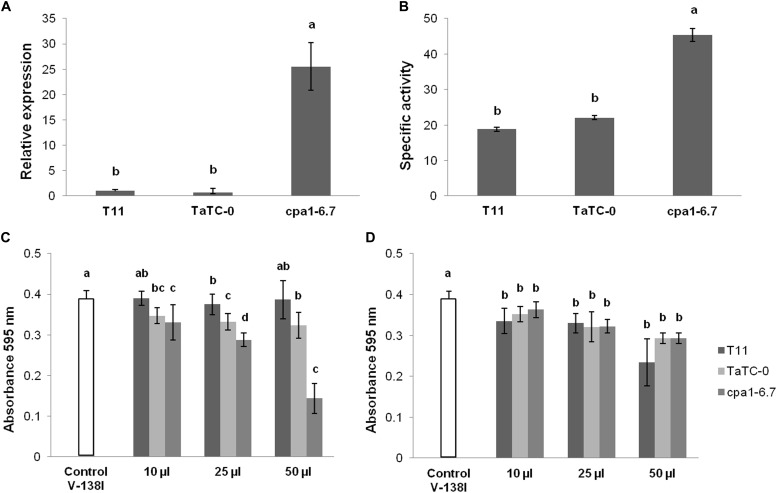
*Trichoderma atroviride* T11 and *cpa1-*overexpressed strains characterization. **(A)** Relative expression of *cpa1* gene transcripts compared with the quantity of the reference gene (actin). Ct values were referred to MM supplemented with 2% glucose as a basal reference. Data are the mean of three biological replicates and are displayed as the change fold differences (RQ, 2^-ΔΔCt^). **(B)** Protease activity measured with 10 μl of protein extracts. Total activity corresponded to mmol of azocasein hydrolyzed in 1 min, and specific activity corresponded to mmol of azocasein hydrolyzed in 1 min per mg of protein. Tests were performed in triplicate and using, at least, three biological replicates, and the data represent mean values with standard deviations. **(C,D)** Antifungal activity measured after 72 h with volumes of 10, 25, and 50 μl of protein extracts unboiled **(C)** and boiled **(D)**. Protein extracts were obtained in all cases from supernatants collected after growing *Trichoderma* strains in MM supplemented with 2% glucose for 24 h. Values with different superscript letters are significantly different according to Tukey’s test (*P* < 0.01).

We analyzed the expression of the *cpa1* gene and protease activity in three independent biological cultures for transformants and wild-type as a reference condition (Figure 3A,B). The transformant *cpa1*-6.7 showed significantly higher *cpa1* transcript levels linked to increased protease activity compared to those detected in T11 or TaTC-0 after 24 h growth in MM supplemented with 2% glucose. The antifungal activity of *T. atroviride* extracellular proteins from these three strains grown in MM supplemented with 2% glucose for 24 h was evaluated against D *V. dahliae* V-138I on 96-well E plates. In these tests, unboiled and boiled protein extracts were considered and hyphal growth from conidia of V-138I was register at 0, 24, 48, and 72 h (Figure 3C,D). Absorbance values recorded at 0 h did not show significant differences among the *T. atroviride* strains assayed for unboiled and boiled conditions. Assays using different quantities of boiled protein extracts from the three *T. atroviride* strains showed no significant differences among them though there was a significant inhibitory effect of all volumes tested (10, 25, and 50 μl) against V-138I at 48 h (data not shown) and 72 h (Figure 3D). Unboiled protein extracts from strain *cpa1*-6.7 were significantly more inhibitory of V-138I than those from T11 or TaTC-0 at 24 (with volumes of 25 and 50 μl, *P* < 0.05), 48 (with volumes of 25 and 50 μl, *P <* 0.01) (data not shown) and 72 h (Figure 3C). Additionally, the larger volume of unboiled protein extracts from strain *cpa1*-6.7 tested the greater inhibitory effect against V-138I was observed (*P* < 0.01). In all cases, quantities (25 and 50 μl) of unboiled protein extracts were positively correlated with the inhibitory effect against V-138I. No significant differences were observed among strains T11, TaTC-0 and *cpa1*-overexpressed transformant on the ability to overgrow a colony of V-138I on MM supplemented with 2% glucose and PDA dual cultures (data not shown).

## Discussion

Verticillium wilt caused by the D *V. dahliae* pathotype is the main soil-borne disease threatening olive production in the Mediterranean Basin (Jiménez-Díaz et al., 2012). In this present work we found that *T. atroviride* T11 has antifungal activity against *V. dahliae* strains representative of races 1 and 2, and D and ND pathotypes in 1A or 2A lineages, respectively (Jiménez-Díaz et al., 2011, 2017). Moreover, results from dual cultures and membrane assays indicate that mycoparasitism and antibiosis seem to be involved in the *T. atroviride* T11 biocontrol activity against *V. dahliae*. Also, T11 appears to bear higher antagonistic potential against *V. dahliae* compared with that reported for *Trichoderma asperellum* strains assayed *in vitro* under conditions identical to those used in this present work (Carrero-Carrón et al., 2016). Assays involving T11 and V-138I on a discontinuous medium showed that release of VOCs, which appears to play a role in communication between both fungi, is also involved in the antagonistic potential of T11 against this pathogen (Supplementary Figure S1).

Previous studies have demonstrated the usefulness of *Trichoderma* HDO microarrays in the analysis of transcriptomic changes in *T. harzianum* under simulated mycoparasitism (Samolski et al., 2009). In the present study, a *Trichoderma* microarray was used to analyze transcriptomic changes that might occur when T11 overgrows colonies of V-138I. The use of an unusual biocontrol target such as *V. dahliae* might serve to gain a deeper insight into the understanding of *Trichoderma* mycoparasitism at molecular level. We have used an experimental design similar to those designed to identify mycoparasitism-related genes in *T. atroviride* IMI 206040 in direct contact with *R. solani* by 454 sequencing (Reithner et al., 2011) and to study mycoparasitic strategies of *T. atroviride*, *T. virens*, and *T. reesei* against *R. solani* (Atanasova et al., 2013) using microarrays. In other studies, liquid cultures and fungal host cell walls were used to identify *T. harzianum* genes expressed under simulated mycoparasitism of *Botrytis cinerea* or *S. sclerotiorum* in microarray (Samolski et al., 2009) or RNA-Seq (Steindorff et al., 2014) approaches.

A high percentage (98.6%) of genes, identified as being differentially expressed in at least two of the three tested conditions, belong to the *T. atroviride* genome. This seems to effectively counteract a common objection made to the use of microarrays regarding propensity to false-positive detections at low expression levels due to cross-hybridizations (Reithner et al., 2011). Moreover, a set of 143 differentially regulated genes was shared in the comparisons of OV with CON and of OV with NV (Table 2 and Supplementary Table S2), and the set of 16 genes used to validate the microarrays displayed a similar expression profile in RTqPCR assays (Figure 2 and Supplementary Figure S2). Thus, these 143 genes would be unequivocally associated with an active antagonism of T11 against V-138I. Among them, the GO categories that were significantly overrepresented are congruent with a mycoparasitic strategy.

When annotation of the 143 *T. atroviride* T11 genes identified was further refined (see Supplementary Table S2) a broad diversity of functions was identified among them, for which a role in mycoparasitism can be inferred. The up-regulation of 21 genes encoding CAZymes from all classes and of 10 protease (serine, aspartic and metallopeptidases) genes in OV is compatible with a mycoparasitic process leading to weakening and hydrolysis of fungal host cell walls, which allows for easier access of T11 to nutrients. A noteworthy increase in expressed CAZyme genes has also been observed in the reported mycoparasitism of *T. harzianum* on *S. sclerotiorum* (Steindorff et al., 2014). By contrast, a down-regulation of several glycosyl hydrolases has been observed in mycoparasitism of *T. atroviride* IMI 206040 on *R. solani* (Atanasova et al., 2013). Also, it has been reported that 52.8% of *T. atroviride* genes were transcribed during self-confrontation, this percentage decreasing to 45% after contact with the fungal host (Reithner et al., 2011). In our study, we have also observed (Figure 2) that some T11 genes were up-regulated in the NT condition and higher expression levels were detected in the OV one. The expression profiles shown in Figure 2 and Supplementary Figure S2 indicate that the level of expression of some genes was affected at a different extent by the proximity of a fungal colony susceptible or not to be parasitized. This agrees with previous reports on the ability of all *Trichoderma* spp. being able to sense other fungi before contact (Seidl et al., 2009; Atanasova et al., 2013).

In this present work, proteolysis was an overrepresented process in condition OV and a peptidases array was consistently up-regulated in this condition, which is in agreement with the significantly higher protease activity detected in OV compared with that in conditions NV and CON. Those results are in line with previous studies that indicated a fundamental role of peptidases in mycoparasitism by *Trichoderma* spp. (Seidl et al., 2009; Atanasova et al., 2013; Steindorff et al., 2014), and with that describing aspartic and serine peptidases are strongly induced by deactivated fungal cell walls as well as by chitin (Suárez et al., 2007). In addition to overexpressed peptidases and CAZyme genes, a notable transporter activity was observed in the OV condition, which was due to the up-regulation of a diversity of transporter genes, with members of the MFS family being the most abundant (Steindorff et al., 2014).

The up-regulation of small secreted cysteine-rich proteins (SCCPs) was expected in the OV condition since these proteins are expanded in the genome of *T. atroviride* (Kubicek et al., 2011) and they have been associated with the mycoparasitism of *Trichoderma* spp. on different phytopathogenic fungi (Omann et al., 2012; Atanasova et al., 2013; Steindorff et al., 2014). Several genes encoding putative proteins related to defense, including two heat-shock proteins and detoxification responses, were up-regulated in OV.

Secondary metabolites enhance mycoparasitism by *T. atroviride* since they act synergistically with cell wall hydrolytic enzymes, thus facilitating the disruption of the host’s structures (Schirmböck et al., 1994; Lorito et al., 2010). Secondary metabolism is enhanced in the mycoparasitism of T11 on V-138I. Thus, it would be expected that secondary metabolites up-regulated in the OV condition and able to inhibit the target fungus would be different from those secreted by T11 growing alone, as indicated by results from membrane assays (Table 1). The presence of secondary metabolites in boiled-protein extracts from all T11 strains would explain their significant inhibitory effects against V-138I considering the absence of protein activities in these samples (Figure 3D). The effect of secondary metabolites in the antagonism ability of *Trichoderma* spp. against fungal pathogens is well documented (Hermosa et al., 2014). One of the most outstanding properties of *T. atroviride* is its enlarged number of genes for the synthesis of polyketide synthases (PKSs) and non-ribosomal peptide synthetases (NRPSs) (Kubicek et al., 2011; Mukherjee et al., 2012). However, of the 20 up-regulated genes related to secondary metabolism in the OV condition only two PKSs (ID211357 and ID32458) and one NRPS (ID156569) were detected out of the 18 and 16 genes annotated, respectively, in the genome of *T. atroviride* (Kubicek et al., 2011). In this present work, numerous up-regulated genes related to primary and secondary metabolic processes, categorized within oxidoreductase activity, were detected in the OV condition. An important set of these genes encoding proteins with binding functions was up-regulated in both *T. atroviride* and *T. virens* during mycoparasitic interactions with *R. solani* (Atanasova et al., 2013). By contrast, other studies have reported repression of this gene category when *T. harzianum* grew in the presence of deactivated cell walls of *S. sclerotiorum* and *Fusarium solani* (Vieira et al., 2013; Steindorff et al., 2014). Two prenyltransferases were also up-regulated in the OV condition, this being compatible with an increase in terpene metabolism. Fungal terpenes are derived from isopentenyl diphosphate building units which cyclization is catalyzed by prenyltransferases to produce mono-, sesqui- and diterpenes that have different antibiotic potential, as well as triterpenes of which ergosterol is the major component of fungal membranes (Liang et al., 2002; Malmierca et al., 2015).

It should be noted that in the OV condition we also observed the up-regulation of three genes encoding isoflavone reductase (IFR)-like proteins with NmrA domains that are involved in the production of isoflavones. In plants, isoflavonoid phytoalexins are key factors in evolutionary processes shaping the rhizosphere microbiome (Philippot et al., 2013). Genes encoding IFRs were up-regulated in *T. atroviride* during contact with *R. solani* (Kubicek et al., 2011; Reithner et al., 2011), and also in *Trichoderma parareesei* in response to presence of tomato plants (Rubio et al., 2014). In these latter studies, IFR genes were functionally related to secondary metabolic processes thought have been also identified as part of a system controlling nitrogen metabolite repression in several fungi (Stammers et al., 2001). The up-regulation of NmrA-like IFR-negative regulators in mycoparasitic processes might indicate the inactivation of pathways for non-preferred nitrogen sources due to the availability of a simpler nitrogen source.

In the present work, we generated *cpa1*-overexpressed transformants as a proof of concept that the set of 143 genes obtained from the microarray analysis are involved in the antagonism of *T. atroviride* T11 to D *V. dahliae* V-138I and its potential of biocontrol against this pathogen. This approach proved to be adequate to analyze genes functionally in *T. harzianum* (Cardoza et al., 2006; Montero-Barrientos et al., 2011). The selection of the *cpa1* gene was based on the overrepresentation of the proteolysis activity in the OV condition confirmed by RTqPCR analysis, as well as on the lack of assigned function in the *T. atroviride* genome JGI-database which made of this gene an interesting candidate to be explored. Studies based on transcriptomic analyses have indicated that proteolysis is a major biological process involved in the mycoparasitism by *Trichoderma* when overgrowing its host (Atanasova et al., 2013; Steindorff et al., 2014). A more recent study has shown that several genes encoding potential blue light photoreceptors are necessary for the appropriate regulation of *cpa1* among others, under exposure to red light (García-Esquivel et al., 2016). The increased protease activity of *cpa1*-overexpressed mutants of *T. atroviride* T11 proved to boost their capacity to inhibit the growth of V-138I in *in vitro* assays (Figure 3C) being this role confirmed by the non-significant effect in boiled-protein extracts (Figure 3D). The induction of the *cpa1* gene was not only triggered when T11 overgrew V-138I but also under the sole presence of inactivated V-138I cell walls. Studies on the characterization of *Trichoderma* proteases have proved that they are involved in mycoparasitism processes. For instance, the *prb1* gene encoding a basic proteinase was induced by presence of phytopathogenic fungi or their cell walls (Geremia et al., 1993), and the aspartic protease P6281 was the most abundant secreted protein in *T. harzianum* cultures growing in oomycete and fungal cell walls (Suárez et al., 2005). Similarly, the expression of four transcripts encoding this same aspartic protease, as well as another aspartic protease together with a serine protease and a M22 metalloprotease of *T. harzianum*, increased significantly after 48 h incubation in *Colletotrichum* cell walls (Sharma et al., 2016). In other studies, T-DNA insertional mutagenesis and UV-irradiation were used to identify genes involved in the ability of *Trichoderma* spp. to antagonize plant pathogens (Szekeres et al., 2004; Zhang et al., 2016). The *nmp1* gene (M35 protease) identified in *Trichoderma guizhouense* plays a role in its antifungal activity against the pathogen *Fusarium oxysporum* f. sp. *cubense* (Zhang et al., 2016), and the overproduction of trypsin-like and chymotrypsin-like proteases in *T. harzianum* increased its antagonistic activity against some fungal plant pathogens (Szekeres et al., 2004). Some studies of high-throughput analysis have identified the regulation of carboxypeptidases under mycoparasitism events (Monteiro et al., 2010; Steindorff et al., 2014). However, carboxypeptidases from the M14 family are poorly characterized proteins although a recent report (Nauom et al., 2018) has identified an hypothetical peptidase M14 gene as differentially expressed in *T. harzianum* cultures supplemented with cell walls of *F. oxysporum* and *S. sclerotiorum*. Results in this present study indicate that CPA1 contributes somehow to the hydrolysis of V-138I cells walls and has a key role in the ability of T11 to successfully inhibit the growth of this fungal pathogen. Nevertheless, the mechanism underlying the activity of this protease needs to be fully explored.

*Trichoderma* spp. contribute to biocontrol of VW caused by *V. dahliae* by means of different mechanisms (Jiménez-Díaz et al., 2012; Carrero-Carrón et al., 2016, 2018), whose efficiency may vary depending upon pathogen strains. Mycoparasitism is an ancestral feature in *Trichoderma* but it is not always displayed in a same way against a given host. Our study provides a further insight in the understanding of the relationships between *T. atroviride* and *V. dahliae*. Strain T11 has shown outstanding biocontrol abilities against different strains of D *V. dahliae* and is able to kill this pathogen by mycoparasitic contact and inhibit its growth through diffusible and VOCs produced before contact. Microarray data of T11 overgrowing colonies of *V. dahliae* indicate that proteolysis is a major biological process involved in mycoparasitism by this strain as proved by the results obtained with T11-*cpa1* mutants. Better understanding of the molecular mechanisms underlying the *T. atroviride-V. dahliae* interaction may allow for increased efficiency in the use of *Trichoderma* spp. for the integrated management of VW in olive and other crops.

## Data Availability

The datasets generated for this study can be found in GEO database GSE66835.

## Author Contributions

RH and EM contributed conception and design of the study. RH, EM, and IC-C analyzed the microarrays raw data and annotated and compared the gene expression data. IC-C, MR, and MM-D participated in the experiments. MM-D performed the statistical analysis. RH, EM, MR, and MM-D drafted the manuscript. RH, EM, and RJ-D contributed to reagents, material, and analysis tools. All authors contributed to manuscript revision, read and approved the submitted version.

## Conflict of Interest Statement

The authors declare that the research was conducted in the absence of any commercial or financial relationships that could be construed as a potential conflict of interest.

## Footnotes

1 http://genome.jgi.doe.gov/Triat2/Triat2.info.html

2 http://genome.jgi-psf.org/Trive1/Trive1.home.html

3 http://genome.jgi-psf.org/Trire2/Trire2.home.html
