# Supplementary material for: Transcriptomic Analysis of Trichoderma atroviride Overgrowing Plant-Wilting Verticillium dahliae Reveals the Role of a New M14 Metallocarboxypeptidase CPA1 in Biocontrol
Source: Front Microbiol. 2019 May 27;10:1120. doi: 10.3389/fmicb.2019.01120 (PMC6545926; doi:10.3389/fmicb.2019.01120)
Supplement: Supplementary file 1 [file Data_Sheet_1.pdf]

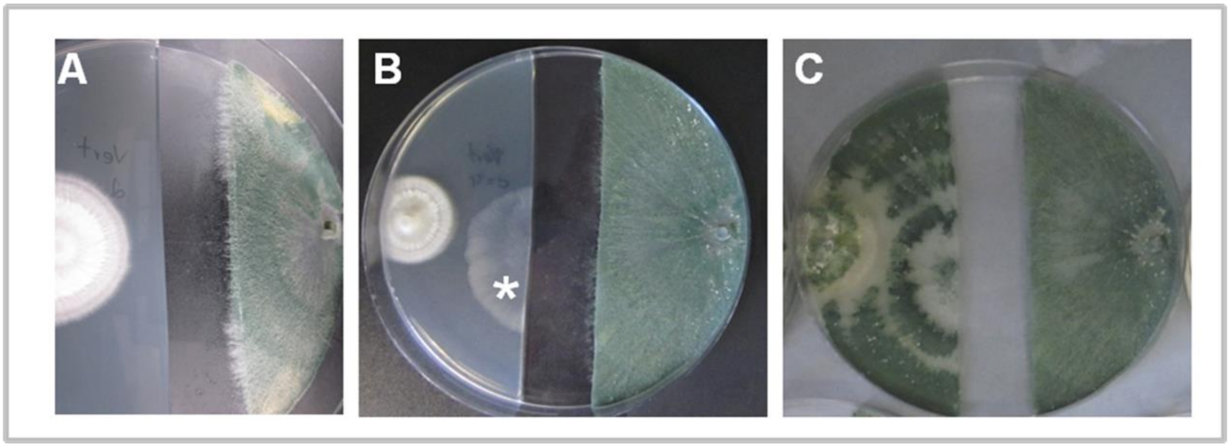

Figure S1. *Trichoderma atroviride* T11 biocontrol profile against *Verticillium dahliae* V-138I on discontinuous agar cultures. **(A)** 7-day colony of T11 confronted with V-138I that was inoculated 4 days in advance. **(B)** 12-day colony of T11 confronted with V-138I where T11 mycelium has already jumped into the left half of the agar plate (*). **(C)** 24-day colony of T11 overgrowing V-138I. Both fungi were inoculated on PDA plates and inoculated at the same time in B and C photos. V-138I was placed on the left and T11 was placed on the right of the images.

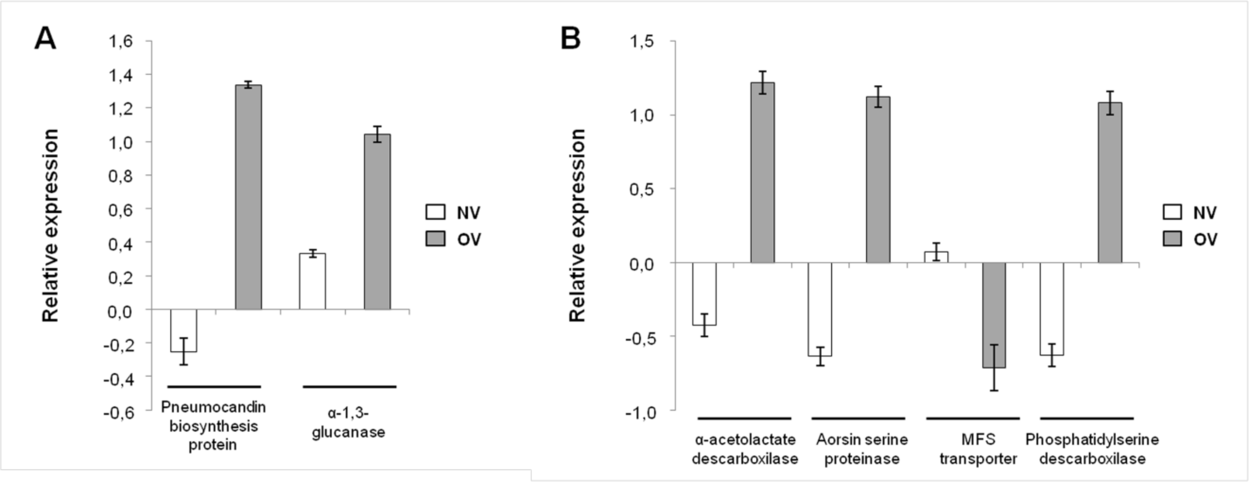

Figure S2. RTqPCR expression analysis of six JGI-referred genes *Trichoderma atroviride* T11 used to validate the microarray data. **(A)** Validation of 2 out of the 18 genes differently expressed when comparing T11 overgrowing *Verticillium dahliae* V-138I (OV) with T11 grown alone (CON). Ct values were referred to the CON condition as a basal reference. **(B)** Validation of 6 out of the 18 genes differently expressed when comparing T11 overgrowing V-138I (OV) with T11 grown at 5 mm from V-138I (NV). Ct values were referred to the NV condition as a basal reference. Genes IDs are assigned according JGI annotation as follows: pneumocandin biosynthesis protein (51260), α-1,3-glucanase (81097), α-acetolactate decarboxylase (48180), aorsin serine proteinase (145909), MFS transporter protein (41962) and phosphatidylserine decarboxylase (146755). Data are the mean of three biological replicates and are displayed as the log10 of the relative quantity (RQ, $2^{-\Delta\Delta Ct}$) of target genes compared with the quantity of actin gene used as a reference.

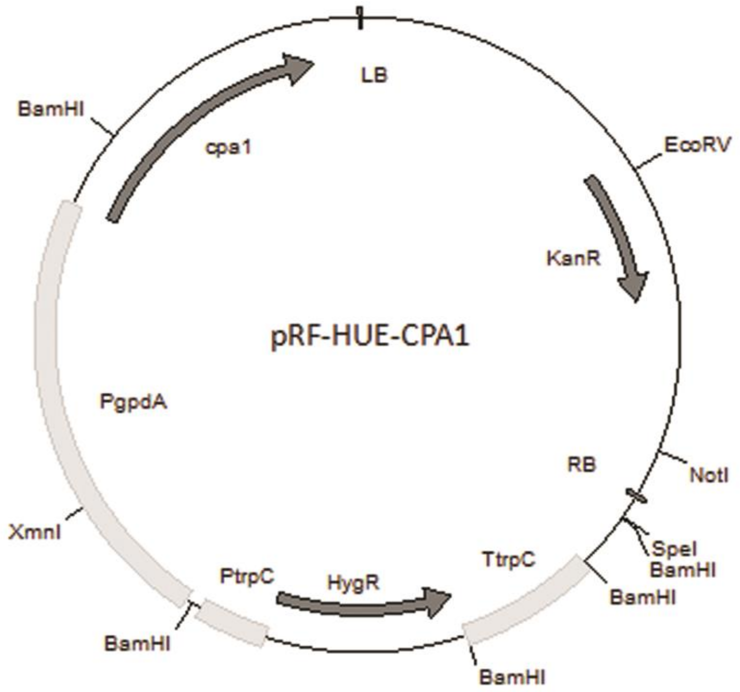

Figure S3. pRF-HUE-CPA1 expression vector for T11 transformation. The pRF-HUE vector-backbone was used for a single cloning step of the *cpa1* gene by the USER™ technology (Frandsen *et al.*, 2008). LB = left border, cpa1 = *cpa1* gene from *Trichoderma atroviride* T11, PgpdA = glyceraldehyde-3-phosphate dehydrogenase promoter from *A. nidulans*, PtrpC = tryptophan promoter from *A. nidulans*, HygR = hygromycin B phosphotransferase, TtrpC = tryptophan terminator from *A. nidulans*, RB = right border, KanR = kanamycin resistance.
