# Supplementary material for: Transcriptomic Analysis of Trichoderma atroviride Overgrowing Plant-Wilting Verticillium dahliae Reveals the Role of a New M14 Metallocarboxypeptidase CPA1 in Biocontrol
Source: Front Microbiol. 2019 May 27;10:1120. doi: 10.3389/fmicb.2019.01120 (PMC6545926; doi:10.3389/fmicb.2019.01120)
Supplement: Supplementary file 2 [file Data_Sheet_2.pdf]

TABLE S1. Specific primer pairs used in RTqPCR analysis

Putative function	JGI ID	Forward (5’-3’)	Reverse (5’-3’)	Slope	Efficiency (%)
Exo-β-1,3-glucanase	48371	ACCAACGATGGTGATGGTCTT	GGTTGCCAGTGCTGGAGATG	-3.24	103.6
Chitinase	52592	ATTCAGCACCCTCAGTCCTGTGGC	GCTGGCAATCAAACTGTGGTTCA	-3.72	85.7
GTP-binding protein	224184	CATTTCTCTTCTCCCGTTTGTTGGC	GGTCAGAGTTGGCGAGGTGCT	-3.86	81.7
Oligopeptide transporter	232557	CTTCGCCTGGATGACGTGGAT	GCGGGAGGGAGTAGGTGG	-3.35	98.7
Acyl-CoA thioesterase 2	282317	CTTTCGAGGGAGATAACGACTG	GGGATTAAGGACAGACACGG	-3.22	104.3
Metalloproteinase	179435	TGGCGATTATATTGCAGATACTCC	TAAATTCAGACCAGCAGCCCT	-3.31	100.7
Cytochrome P450 monooxygenase	295844	AATGCGCTCTACGATGTTTATGG	CATGTTGCACCTTGTTACTGAG	-3.36	98.5
Alkaline proteinase	302419	GCGGGCGAGTTTGACGAGG	CCCAAGGAGCACCAGATTGTGTG	-3.06	112.5
Zinc carboxypeptidase A1	301733	CGCACATTCATCAACGGCAACA	CCTCTTTGACCTGGGCGTTGG	-3.26	102.4
FAD-monooxygenase	32449	GCTGATAAGTTTCCACGATACC	AAATCTGTATATCCTTCCCGCT	-3.33	99.5
Pneumocandin biosynthesis protein	51260	CTCAATGGCTTGTCAGATTCGT	AGACTGTTGAAGAATACACCCTG	-3.53	92.1
α-1,3 glucanase	81097	CATTTCATGATCGGCATTGTTGG	GCGTATTGAGCAATCTTCTGG	-3.37	98.0
α-acetolactate decarboxylase	48180	GCCACGGGAACCACGGCCT	GACGGAGGGCTGGAAGGAGGT	-3.17	107.0
Aorsin serine proteinase	145909	CTATCTCGAGCGCCAATGTG	GTACTGGCTTGGTAGCTTGG	-3.31	100.3
MFS transporter	41962	CAAAGTTCTCAACTGCGTCCT	GATGACGAAGACGATGAAGCC	-3.55	91.39
Phosphatidylserine decarboxylase	146755	AATCTTGGGACGACTTCTTTACAC	AATGCCTGATAAACTGTTGCTCC	-3.48	94.0
Actin		ATCGGTATGGGTCAGAAGGA	ATGTCAACACGAGCAATGG	-3.46	94.6

TABLE S2. Functional distribution and hit description of the 143 differentially expressed genes during the overgrowth of *Trichoderma atroviride* T11 on *Verticillium dahliae* V-138I

Functional distribution	JGI ID	Hit Description	E-value
METABOLISM			
Carbohydrate	32968	Thaumatin, pathogenesis-related	0.0
	33348	Endo-β-1,3-glucanase	$4e^{-122}$
	37969	Endo-β-1,3-glucanase	$3e^{-168}$
	41194	Endo-β-1,3-glucanase	0.0
	46148	Six-hairpin glycosidase	$1e^{-143}$
	48371	Exo-β-1,3-glucanase	$1e^{-131}$
	52592	Chitinase	$5e^{-82}$
	54743	Exo-β-1,3-glucanase	$4e^{-56}$
	217415	Glycoside hydrolase family 18	0.0
	224184	Thaumatin-like protein	$5e^{-23}$
	161159*	β-xylosidase	0.0
	42346	Carbohydrate esterase family 1 protein	0.0
	79361	Carbohydratase-esterase family 9 protein	0.0
	302304	Cutinase	$1e^{-127}$
	82388	Chain A, crystal structure of endo-β-1,4 glucoronan lyase	$4e^{-125}$

	77441	Glycosyltransferase	0.0
	184052	Glycosyltransferase	$4e^{-165}$
	288740	Glycosyltransferase	$2e^{-170}$
	224400*	Glycogen debranching enzyme	0.0
	37996	Short chain dehydrogenase/reductase	$9e^{-57}$
	90220	Dehydrogenase	$1e^{-106}$
	217448	Short chain dehydrogenase/reductase	$1e^{-111}$
	275833	Short chain dehydrogenase/reductase	$5e^{-69}$
Lipid and fatty acid	43454	Thioesterase superfamily protein	$1e^{-30}$
	282317	Acyl-CoA thioesterase 2	$2e^{-126}$
	322224	1 H-3-hydroxy-4-oxoquinaldine 2,4-dioxygenase	$4e^{-67}$
	90126	AMP-dependent CoA ligase	0.0
	229608	Long-chain-fatty-acid-CoA ligase	$1e^{-167}$
	43297	Enoyl-CoA hydratase/isomerase	$2e^{-144}$
	39628	Glycerol kinase	0.0
	140671*	Methyltransferase type 11	0.0
Protein	36337	Deuterolysin metallopeptidase	$3e^{-92}$
	54382	Dipeptidyl-peptidase	$1e^{-147}$
	168477	Metallopeptidase	$2e^{-91}$
	179435	Metalloprotease	0.0
	227276	Subtilisin protease	0.0

	288311	Subtilibsin protease	0.0
	292296	Aspartic endopeptidase	0.0
	299629	Fungalysin metalloproteinase	0.0
	301733	Zinc carboxypeptidase A1	0.0
	302419	Alkaline proteinase	0.0
Amino acid	30776	D-amino acid aminotransferase	$5e^{-145}$
	93352	Cysteine synthase	0.0
	52932	AMP-binding enzyme	0.0
	260926	Homoserine o-acetyltransferase	$1e^{-167}$
Nucleic acid	35700	Bacterial-type extracellular deoxyribonuclease	$5e^{-80}$
	314350	Dihydrofolate reductase	$3e^{-117}$
Secondary	42070	Isoamyl alcohol oxidase	$5e^{-151}$
	43092	Oxidoreductase (related to flavine oxidoreductase)	$3e^{-148}$
	43714	Manitol dehydrogenase	$3e^{-134}$
	48755	Indoleamine 2,3-dioxygenase	0.0
	84122	L-aminoadipate-semialdehyde dehydrogenase	$1e^{-168}$
	210776	Polyamine oxidase	0.0

	314346	Lactate dehydrogenase	$7e^{-171}$
	45759*	Clavaminate synthase	$2e^{-166}$
	280794*	Chloroperoxidase	$7e^{-58}$
	32458	Polyketide synthase	0.0
	211357	Polyketide synthase	0.0
	156569	Nonribosomal peptide synthetase	0.0
	32449	FAD-monooxygenase	0.0
	52551	L-lysine 6-monooxygenase/L-ornithine 5-monooxygenase	$8e^{-153}$
	250440	Cyclohexanone monooxygenase	$2e^{-142}$
	42245	Ubi A prenyltransferase	$5e^{-88}$
	295847	Prenyltransferase	$1e^{-49}$
	37750	Trichothecene C-15 hydroxylase	$4e^{-122}$
	43598	Cytochrome P450	0.0
	84128	Cytochrome P450 monooxygenase	0.0
	295844	Cytochrome P450 monooxygenase	0.0
	90918	Zn-dependent hydrolase/oxydoreductase family	0.0
Energy	46393	Dehydrogenase	$2e^{-103}$
	53082	NADP-dependent leukotriene b4 12-hydroxydehydrogenase	$1e^{-179}$
	162830	α–ketoglutarate-dependent sulfonate dioxygenase	0.0
	239450	NAD(P)-binding	$4e^{-77}$

	221344	Haloacid dehydrogenase	$7e^{-73}$
	288362*	Phosphoglycerate mutase	$4e^{-89}$
CELLULAR PROCESSES AND SIGNALING			
Transport	129432	Oligopeptide transporter	0.0
	232557	Oligopeptide transporter	0.0
	53012	MSF transporter	0.0
	81541	Efflux pump antibiotic resistance protein	0.0
	84436	Rivoflavin transporter	$6e^{-151}$
	262763	Flavin-nucleotide binding	$1e^{-118}$
	251581	Tetracycline resistance protein	$5e^{-132}$
	280284	MSF transporter	$3e^{-31}$
	229348	Cytosine-purine permease	0.0
	139683	Globin-like-protein	$4e^{-133}$
	284539	Cobalamin (Vitamin B12) domain	0.0
	81501	Transmembrane aminoacid transporter	0.0
	296589	Dicarboxylic amino acid permease	0.0

	290277	GPR1 family protein	$7e^{-71}$
Defense	299986	Heat shock protein (hsp23)	$2e^{-75}$
	301737	Heat shock protein (hsp20)	$3e^{-120}$
	85009	Mss4-like protein	$2e^{-60}$
	77850	Gluthatione S-transferase	$2e^{-79}$
	142741	Glycolate oxidase	0.0
	37150	Acetyltransferase	$4e^{-128}$
Signaling	290919	Small secreted cysteine-rich protein	0.0
	298730	Small secreted cysteine-rich protein	0.0
	TriviG29_70385**	Small secreted cysteine rich protein	0.0
	283740*	Small secreted cysteine-rich protein	0.0
	300292*	GPR1 family	$3e^{-144}$
	239712	Phosphatidylinositide-specific phospholipases C	0.0
Cell-cell contact and communication	294485*	Rearragement hotspot protein	0.0
	269934	Tol protein	$3e^{-42}$
Detoxification	278807	Tat pathway signal sequence	$2e^{-37}$
Cell wall and membrane	46317	PTH-11 integral membrane	$3e^{-96}$
	81348	Expansin module family	$3e^{-76}$
Regulation	41299	Related to cercosporin resistance	$2e^{-145}$
	164928	Homeobox transcription factor	$2e^{-82}$

	301901	STF2 protein	$3e^{-41}$
Postranslation events	256937	Isoflavone reductase-like protein	$7e^{-66}$
	302964	NmrA-like family protein	$4e^{-27}$
	88236	NmrA-like family protein	$6e^{-80}$
INFORMATION STORAGE AND PROCESSING			
Transcription	90244	Methyltransferase	$4e^{-58}$
Translation	131910	Valyl-tRNA synthetase mitochondrial precursor	0.0
	246441	Amidase	0.0

*: down-regulated genes

**: gene identified from probe designed against *T. virens* genome

TABLE S3. Functional assignation of 18 differentially expressed genes [Fold-change (FC) > 2, FDR < 0.15, *P*<0.05] occurring only between *Trichoderma atroviride* T11 overgrowing *Verticillium dahliae* V-138I and T11 grown alone

Functional distribution	Hit description (E-value < 10^{-20} limit)	Lg_2 FC	JGI ID
METABOLISM			
Carbohydrates	Short-chain dehydrogenase/reductase	4.69	306768
	β-1,3-glucanase	2.44	315637
	α-1,3-glucanase	2.02	81097
Secondary	Taurine catabolism monooxygenase	-2.43	46162
	Tyrosine metabolic process	-2.35	63940
	Pneumocandin biosynthesis	2.12	51260
	Cytochrome P450 monooxygenase	2.08	263459
CELLULAR PROCESSES AND SIGNALING			
Transport	MFS transporter	2.38	30874
	Purine permease	2.25	42539

	Allantoate permease	2.23	46082
Defense	Heat-shock protein 78	2.17	157172
Signaling	G-protein-coupled receptor	2.08	130836
Detoxification	Cytochrome oxidase assembly protein	4.44	84409
Regulation	Fungal transcriptional regulatory protein	2.07	321925
Adhesion	Fibronectin	-2.64	299265
UNKNOWN FUNCTION			
	-	2.10	171019
	-	2.01	302901
	-	-2.01	300321

*Identification (ID) in the *T. atroviride* genome [Joint Genome Institute (JGI)].

TABLE S4. Functional assignation of 18 differentially expressed genes [Fold-change (FC) > 2, FDR < 0.15, P<0.05] occurring only between *Trichoderma atroviride* T11 overgrowing *Verticillium dahliae* V-138I and T11 at ca 5 mm of V-138I

Functional distribution	Hit description (E-value < 10^{-20} limit)	Lg_2 FC	JGI ID*
METABOLISM			
Carbohydrates	α-acetolactate decarboxylase	2.16	48180
	Aorsin (serine proteinase)	2.10	145909
Lipids	Phosphatidylserine decarboxylase	2.11	146755
	3-β-hydroxysteroid dehydrogenase	2.01	214542
Nitrogen	NAD(P)H Azoreductase	2.43	297537
Secondary	Chanoclavine synthase	-2.04	152289
CELLULAR PROCESSES AND SIGNALING			
Transport	MFS transporter	-2.97	41962
	MFS transporter	-2.85	42539
	Monocarboxylate transporter	2.29	296804

	Fe/Mn superoxide transporter	2.12	48025
Signaling	Ankyrin repeat protein	2.34	181516
Adhesion	Protein with von Willebrand factor A (VWA)-like modules	2.11	213544
UNKNOWN FUNCTION			
	-	-2.87	77767
	-	-2.29	297301
	-	2.17	49033
	-	2.10	35776
	-	2.03	185735
	-	2.02	49033

*Identification (ID) in the *T. atroviride* genome [Joint Genome Institute (JGI)].
